# When the Secondary Survey is Primary: Knife Blade in the Spine

**DOI:** 10.5811/westjem.2015.9.28642

**Published:** 2015-12-01

**Authors:** Lauren M. Porter, Robert D. Barraco, Stephanie Goren-Garcia, Jeanne L. Jacoby

**Affiliations:** * Lehigh Valley Health Network, Department of Emergency Medicine, Allentown, Pennsylvania; † Lehigh Valley Health Network, Department of Surgery, Allentown, Pennsylvania; ‡ University of South Florida Morsani College of Medicine, Tampa, Florida

A 42-year-old male was assisted from a car in front of our inner city stand-alone emergency department (ED) with a stab wound to the right chest. He was confused and bleeding; his past medical history was unknown. The patient was diaphoretic, pale and confused with a large vertical stab wound over his right chest with no other obvious injuries. On initial exam in the outlying ED, his back was obscured by blood. He was transferred to the trauma center where during a full secondary survey a 2cm wound was located over the patient’s lumbar spine. The patient was stabilized and taken for imaging. No focused assessment with sonography for trauma (FAST) was done at either site; however, the FAST exam, which emphasizes the search for extraluminal blood, would not have been expected to find a foreign body. Computed tomography (Figure) showed a retained 10cm blade extending through the left L1-L2 interlaminar space, spinal canal and disc space with associated injuries to the IVC and renal vein, duodenum and medial left hepatic lobe. On day one he had two damage control laparotomies. The blade could not be removed due to patient instability and inability to turn him prone. On day two, he returned to the operating room and the blade was removed by resecting the spinous process and lamina of L1 with joint efforts by the vascular, neurosurgery and trauma surgeons. He required five additional surgeries to wash out and finally close his abdomen and was discharged to home after 39 days. He was neurologically intact.

Spinal stab wounds with retained knife blades are uncommon in the U.S. A literature search revealed few case reports.^1^–^3^ Enicker et. al present a 12-year case series from South Africa where a majority had neurologic deficits consistent with Brown-Sequard syndrome.^4^ A U.S. case report presented a patient who presented four weeks after an initial stab wound to the spine with worsening neurologic deficits necessitating knife fragment removal.^5^ The importance of a complete secondary survey in the evaluation of assault victims in order to detect spinal injuries and possibly prevent neurologic sequelae is demonstrated in this case.

## Figures and Tables

**Figure:**
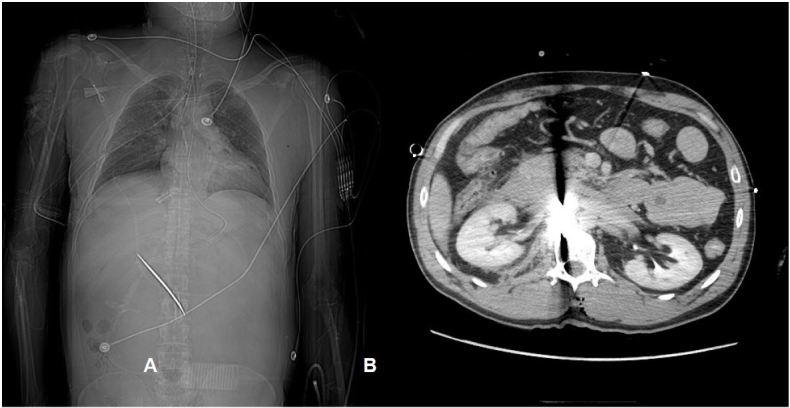
A) Scout film demonstrating retained knife blade. B) computed tomography demonstrating the retained knife blade and resulting artifact.
